# Experience of Primary Care among Homeless Individuals with Mental Health Conditions

**DOI:** 10.1371/journal.pone.0117395

**Published:** 2015-02-06

**Authors:** Joya G. Chrystal, Dawn L. Glover, Alexander S. Young, Fiona Whelan, Erika L. Austin, Nancy K. Johnson, David E. Pollio, Cheryl L. Holt, Erin Stringfellow, Adam J. Gordon, Theresa A. Kim, Shanette G. Daigle, Jocelyn L. Steward, Stefan G Kertesz

**Affiliations:** 1 Mental Illness Research, Education, and Clinical Center (MIRECC), Veterans Affairs Greater Los Angeles Healthcare System, Los Angeles, California, United States of America; 2 University of California Los Angeles, Department of Psychiatry and Biobehavioral Sciences, Los Angeles, California, United States of America; 3 Center for Surgical, Medical Acute Care Research and Transitions (C-SMART), Birmingham Veterans Affairs Medical Center, Birmingham, Alabama, United States of America; 4 University of Alabama, School of Social Work, Tuscaloosa, Alabama, United States of America; 5 University of Maryland, Department of Behavioral and Community Health, College Park, Maryland, United States of America; 6 Boston Health Care for the Homeless Program, Boston, Massachusetts, United States of America; 7 Veterans Affairs Pittsburgh Healthcare System, Pittsburgh, Pennsylvania, United States of America; 8 University of Pittsburgh, School of Medicine, Pittsburgh, Pennsylvania, United States of America; 9 Boston Medical Center, Boston, Massachusetts, United States of America; 10 Boston University, School of Medicine, Boston, Massachusetts, United States of America; 11 University of Alabama at Birmingham, School of Medicine, Birmingham, Alabama, United States of America; Supportive care, Early DIagnosis and Advanced disease (SEDA) research group, UNITED KINGDOM

## Abstract

The delivery of primary care to homeless individuals with mental health conditions presents unique challenges. To inform healthcare improvement, we studied predictors of favorable primary care experience among homeless persons with mental health conditions treated at sites that varied in degree of homeless-specific service tailoring. This was a multi-site, survey-based comparison of primary care experiences at three mainstream primary care clinics of the Veterans Administration (VA), one homeless-tailored VA clinic, and one tailored non-VA healthcare program. Persons who accessed primary care service two or more times from July 2008 through June 2010 (N = 366) were randomly sampled. Predictor variables included patient and organization characteristics suggested by the patient perception model developed by Sofaer and Firminger (2005), with an emphasis on mental health. The primary care experience was assessed with the Primary Care Quality-Homeless (PCQ-H) questionnaire, a validated survey instrument. Multiple regression identified predictors of positive experiences (i.e. higher PCQ-H total score). Significant predictors of a positive experience included a site offering tailored service design, perceived choice among providers, and currently domiciled status. There was an interaction effect between site and severe psychiatric symptoms. For persons with severe psychiatric symptoms, a homeless-tailored service design was significantly associated with a more favorable primary care experience. For persons without severe psychiatric symptoms, this difference was not significant. This study supports the importance of tailored healthcare delivery designed for homeless persons’ needs, with such services potentially holding special relevance for persons with mental health conditions. To improve patient experience among the homeless, organizations may want to deliver services that are tailored to homelessness and offer a choice of providers.

## Introduction

Persons experiencing homelessness are faced with medical, social, and environmental challenges to their physical and mental health. Moreover, persons experiencing homelessness disproportionately suffer from medical illness, problematic substance use, and psychiatric disorders [1–2]. High rates of psychiatric disorders within homeless populations can make health management access and utilization more complex [3–4]. For instance, persons with mental illness access primary care less frequently than patients without mental illness, yielding worse chronic disease management outcomes, and disproportionately high premature mortality rates [5].

For homeless individuals, these challenges are compounded by health service underutilization with significant unmet needs and problems accessing primary and specialty care, [6–8] in turn increasing use of hospital and emergency departments [9–11]. Even when payment is assured, as in Canada [8, 12] or the Veterans Health Administration, [13] this remains the case.

Efforts to remediate these challenges include initiatives to extend the patient-centered medical home (PCMH) concept to homeless individuals. As summarized by Strange et al., the PCMH embodies core principles of primary care (access, comprehensiveness, integration, and relationship), new ways of organizing practice and its payment, and attention to a wide range of performance indicators in which patients play a crucial role in reporting whether key attributes of primary care are in fact being attained [14].

In recent years, both the US Department of Veterans Affairs and the US Department of Health and Human Services have recently advanced PCMH models for persons experiencing, or emerging from, homelessness. The Department of Veterans Affairs funded 37 such programs starting in 2012, [15] and their use appears to facilitate reductions in emergency department services [16]. The Health Care for the Homeless Program, operating with the US Department of Health and Human Services (HHS), has long included strong examples of this nature, [17] and HHS has strongly encouraged the adoption of the PCMH model by federally qualified health centers, and advanced a special initiative multiply diagnosed HIV positive homeless individuals [18].

By definition, patients’ assessments of their experience in care serve as crucial indicators of success in fostering a PCMH, [14] and their positive assessments correlate with retention, adherence and improvements in some conditions [19–21]. For homeless patients with mental health conditions in particular, there is ample qualitative evidence that seeking care can be an adverse experience colored by stigma, and lack of coordination or disrespect [22]. Research efforts to systematically understand what patient or system characteristics help to produce a favorable care experience for homeless patients with mental health conditions are scarce. The present study utilized a new patient-reported 33-item instrument developed specifically to capture homeless persons’ experience of primary care, the Primary Care Quality-Homeless survey [23]. Drawing on data from a sample of patients receiving care at 5 sites across the country, we sought to identify determinants of primary care experiences for homeless persons with mental health conditions across various delivery models.

## Methods

### Design

We evaluated predictors of a more favorable primary care experience across various care settings for homeless persons with mental health conditions. This is an analysis of data from a larger study that developed and validated a survey, the Primary Care Quality—Homeless (PCQ-H-33), designed to assess perceptions of health care in homeless individuals [23]. This survey was read aloud to each participant by research associates at five sites, and took 5–10 minutes to complete. Procedures were approved by the Institutional Review Boards represented at each of the five sites.

### Sites

The five sites differed in the extent to which they were tailored for homeless care services. Homeless-specific tailoring is thought to exist along a continuum [23–24]. The most tailored environments have dedicated program staff, specialized training of staff, heavy emphasis on walk-in availability, the capacity to respond to tangible or competing needs (such as food, washing or clothing), integrated mental health care, and inclusion of homeless individuals in organizational governance. Two of the five sites (Tailored VA and Tailored Non-VA) overtly tailored primary care service delivery. The Tailored VA site was designed and funded specifically for homeless patients, and included co-located mental health and primary care with an emphasis on access and same-day services [25]. The Tailored Non-VA site had the most homeless-centric service characteristics, providing outreach care within the community, homeless-focused medical and nursing staff, as well as representation of homeless and formerly homeless persons in organizational governance [17]. The remaining three sites (VA-A, VA-B, VA-C) offered mainstream primary care operations within standard VA clinic settings serving homeless and non-homeless persons alike. Among these, one site (VA-A) had a component of service tailoring in that a minority of patients received primary care in shelters or a VA domiciliary, although most did not.

### Participants

Participants, including veterans and non-veterans, were obtained by randomly sampling patients at each site who met criteria for (1) presumptive past or current homelessness, and (2) receipt of primary care two or more times in the past two years. In the four VA sites, presumptive past or current homelessness was based on an International Classification of Diseases-9-CM code of V60.0 diagnosis. In the tailored non-VA site, past or current homelessness was based on utilization of the site for care. Across all five sites, 6371 persons met criteria for two primary care visits between July 2008 and June 2010. Of these, 2584 (41%) were selected for recruitment, 870 (14%) were contacted and screened, and 634 (10%) entered the study [23]. For analyses, 366 were selected based on having mental health conditions. Inclusion criteria for mental health conditions were one or more of the following: (1) diagnosis of PTSD or schizophrenia, (2) history of receiving psychiatric medication (“had a significant period of time in which medication was prescribed for any psychological or emotional problems”), or (3) endorsed severe psychiatric symptoms based on a 14-item Colorado Symptom Index (CSI) using a score cut-off of 30 or greater [26–27]. Diagnosis and psychiatric medication status were self-reported. Previously published examination of psychometric properties of the CSI found that a score of 30 has respectable sensitivity (.76) and specificity (.68) and that the CSI is “fair to good” discriminator of individuals with psychiatric disabilities [28]. Given the higher prevalence of such disorders among the homeless, the positive predictive value of a score over 30 is likely to be high.

## Measures

### Conceptual model

**Fig 1:**
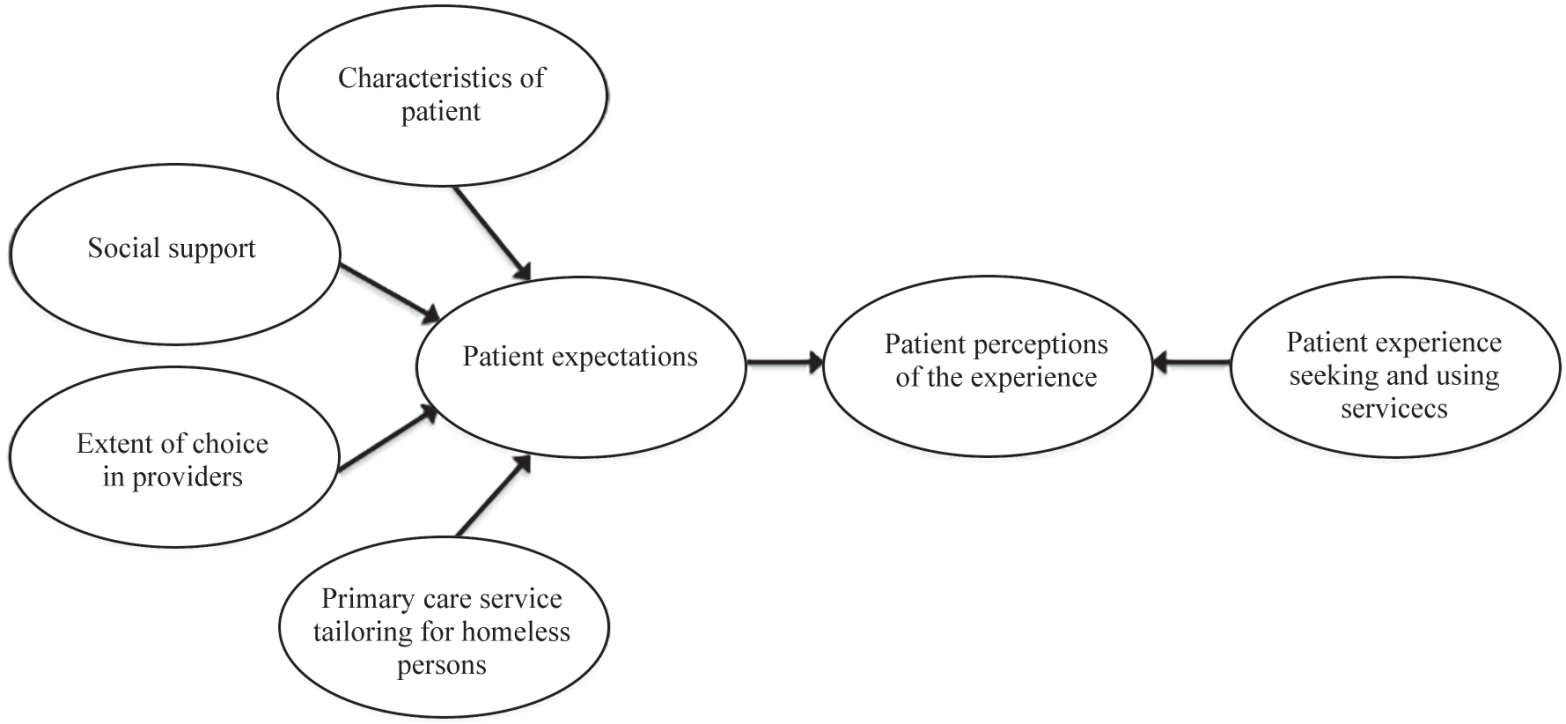
Adapted conceptual framework for predictors of patient care experiences among individual experiencing homelessness. This framework was adapted from Shosanna Sofaer and Kirsten Firminger model of patient perceptions. For the purpose of our analysis, we retained some original variables as well as the general relationship expressed in this model, although the variables are specific to our sample population.

Sofaer and Firminger’s patient perceptions model guided choice of predictors related to primary care experience [29]. As shown in Fig. 1, nodes within the model include the following measured variables: characteristics of patient (demographics, health, depression/anxiety, severe psychiatric symptoms, drug and alcohol severity, and housing status), social support, perceived extent of choice among providers, primary care service tailoring for homeless persons, and patient experience seeking and using services. Within this model, the nodes highlight the predictive relationship between the nodes and outcome variable, patient perceptions of the primary care experience. In modifying Sofaer and Firminger’s model, we added two particular nodes of interest: social support and primary care service tailoring for homeless persons (based on receiving care at either of two tailored primary care programs, compared to three mainstream programs). Recent studies have highlighted the importance of both variables in similarly vulnerable populations [30–31]. Our adaptation of the model is intended to be conceptually broad and generalizable to other vulnerable populations. However dimensions within the adapted model highlight important characteristics in populations with mental health conditions.

### Covariates

Potential predictive factors related to homeless persons’ experience in primary care were construed as follows: characteristics of patient, social support, perceived extent of choice in providers, primary care service tailoring for homeless persons, and patient experience seeking and using services. Characteristics of patients included demographics such as age, gender, race, marital status, education, employment, and income, as well as health, depression/anxiety, severe psychiatric symptoms, drug severity, alcohol severity, and housing status.

Health was measured by the single-item General Self-Reported Health question, which strongly predicts both mortality and health care utilization [32–33]. Subscales for depression/anxiety, and severe psychiatric symptoms were devised from items within the CSI, using items that scaled distinctly in published factor analysis [28]. For depression/anxiety, a score was computed from the sum of five items regarding feelings in the past month (e.g., "nervous, tense, worried, frustrated, and afraid," "depressed," "lonely," "trouble making up mind," and "trouble concentrating"). For severe psychiatric symptoms, a score was computed from the sum of two items regarding feelings in the past month (e.g., "others suggested you acted paranoid/suspicious,” and “hear voices/see things other people didn’t think were there”) [27]. Both items are often associated with psychotic disorders, although other mental health conditions can generate similar symptoms. Drug and alcohol severity was assessed using the Global Continuum Illicit Drug Use score, and the Specific Current Alcohol Involvement score from the Alcohol, Smoking and Substance Involvement Screening Test (ASSIST) [34]. Housing status was devised into four categories: recently and chronically homeless, recently homeless without a history of chronic homelessness, chronically homeless, and neither chronically or recently homeless. Chronic homelessness refers to four or more episodes in the past three years, or a period longer than one year [35–36]. Recent homelessness refers to any nights spent on the street or in a shelter in the last six months [37]. Social support was devised from Lin and Dean’s Strong Ties scale comprised of three items (e.g., “Not having a close companion,” “Not having enough friendships,” and “Not seeing enough of the people you feel close to: 1 = most/all of the time, 2 = occasionally, 3 = sometimes, 4 = rarely, 5 = never”) [38]. Scores ranging from 3 suggest low companionship, and scores ranging to 15 suggests high companionship. Extent of choice in providers was defined as a patient’s perceived choice of provider measured on a 4-point Likert scale (e.g., “I can switch primary care doctors if I ask: 1 = strongly disagree; 2 = disagree; 3 = agree; 4 = strongly agree; 99 = NA”). Primary care service tailoring was examined along a continuum that varied in the degree of homeless-tailored service design, from none (i.e., “mainstream primary care”) to intensive tailoring [23]. Utilization of hospital and emergency department use in the last six months was self-report (e.g., “How many times did you visit a medical clinic or private doctor,” “How many times have you been to a substance abuse counselor in an outpatient program,” “How many nights did you spend in a hospital in order to receive care for yourself,” and “How many nights did you visit the emergency room or urgent care clinic for medical care?”). Self-report of whether a major health care utilization event occurred has been shown to be reasonably valid, [39] including in samples with mental disability, and represents a less-biased estimator for case-mix control [40].

### Outcomes

The primary dependent variable, the overall perception of primary care experience, was measured using a recently validated patient-reported survey, the Primary Care Quality—Homeless (PCQ-H) Survey. The survey was developed from qualitative interviews and focus groups, item drafting by a multidisciplinary team, and psychometric analysis using Item-Response Theory [23]. The resultant 33-item PCQ-H Survey permits computation of a single overall score (“overall PCQ-H score”), the primary outcome of this analysis. It also generates four scale scores related to: 1) Patient-clinician relationship, 2) Perceived cooperation among clinicians, 3) Accessibility/coordination, and 4) Homeless-Specific Needs, but these are not a focus of the present manuscript. Though the single scale is of interest to us here, both the single scale, and 4-scale solutions produce good fit to data (Comparative Fit Index 0.924 for the single-scale, and 0.936 for the 4-scale solution).

### Statistical methods

A general linear model was used to assess predictors of overall primary care experience drawing upon the Sofaer and Firminger perceptions of care model [29]. Model variables included demographics (age, gender, years of education, race), patient characteristics (depression/anxiety symptoms, severe psychiatric symptoms, drug severity, alcohol severity, health, housing status), and other factors (social support, perceived choice among providers based on a Likert-type item, site of care) including the interaction of severe psychiatric symptoms with site. To aid in the understanding of results, some variables were dichotomized: health was split into excellent/ very good/ good versus fair/ poor. The variables that had a high zero-inflation (drug and alcohol severity, and severe psychiatric symptoms) were dichotomized into presence and absence of symptoms. Also, to further explore the interaction of severe psychiatric symptoms by site, a separate general linear model was run for presence/absence of such symptoms.

A likelihood ratio test was used to compare a model with demographics, patient characteristics and other factors to a simpler model with patient characteristics and other factors. Analyses were conducted using Statistical Software Package SAS Version 9.1.3.

## Results

The majority of the sample was male (83%). With regard to race, 33% were White, 56% African-American, 2% American Indian/Alaskan Native, <1% Asian/Pacific Islander, and 9% other race. Mean age was 52 years old (± 16), years of education were 12.9 (SD = 2), 82% of the sample had an annual income of less than $15,000 (Table 1). The mean PCQ-H score for all persons included in the analysis was 3.13 (SD = 0.38) on a 4-point Likert Scale.

**Table 1:** PCQ-H sample population demographics across five clinic sites.

Total sample	366	-
Demographics		
Age (years)	343	51.8 (8.5)
Gender		
Male	303 (82.8)	-
Female	59 (16.1)	-
Other/Transgender	4 (1.1)	-
Single/Divorced	280 (76.9)	-
Employment		
Full or Part time	52 (14.3)	-
Unemployed	97 (26.7)	-
Disabled	164 (45.2)	-
Other	50 (13.8)	-
Race		
White	120 (32.8)	-
African American	204 (55.7)	-
American Indian/Alaskan Native	9 (2.5)	-
Asian/Pacific Islander	1 (0.27)	-
Other	32 (8.74)	-
Income		
< $15,000 per year	270 (82.1)	-
$16,000 or more	59 (17.9)	-
Education (years)	364	12.9 (2.0)
Utilization		
Number of times medical clinic or a private doctor (past 6 months)	366	7.5 (15.1)
Number of times substance abuse counselor in an outpatient program (past 6 months)	365	9.0 (24.8)
Nights in hospital (past 6 months)	366	3.2 (12.1)
Emergency room or urgent care clinic for medical care (past 6 months)	366	0.9 (1.9)

The likelihood ratio test determined that demographic variables did not increase predictive validity and were therefore excluded from the model χ^2^ = -5.5, df = 7, p < 0.05. The linear combination of the remaining predictive factors was significantly related to the primary care experience score, *F* (21, 354) = 8.95, *p* ≤0.0001 and accounted for approximately 36% of the variation in the sample. In Table 2, we present the individual predictors. The relationships between the predictive measures and the primary care experience score were in the expected direction; site of care with tailored sites and Mainstream A (the one with some tailoring) obtaining more favorable scores (F = 2.80, p = 0.03), with perceived extent of choice (F = 23.29, p<0.0001), and housing status (F = 2.91, p = 0.03), proving significant. There was a significant interaction of severe psychiatric symptoms by site [F(df = 4x1) = 3.61, p = 0.01)], further treated below. Non-significant covariates included depression/anxiety (F = 1.79, p = 0.18), severe psychiatric symptoms (F = 2.39, p<0.12), drug severity (F = 0.81, p<0.37), alcohol severity (F = 0.01, p<0.93, and social support (F = 0.77, p<0.38). General health status approached but did not attain significance (F = 3.38, p≤0.07).

**Table 2:** The effect of patient and site characteristics on predictors of care experiences among PCQ-H sample population ^**1**^.

	F Value (p value)	Mean (SE)	Estimate (SE)	t Value (p value)
Site of Care	F = 2.80 (p = 0.03)	-	-	-
Tailored Non-VA (Massachusetts)	-	3.14 (0.05)	-	-
Tailored VA (California)	-	3.05 (0.06)	0.04 (0.09)	0.44 (0.66)
Mainstream VA-A (Pennsylvania)	-	3.06 (0.05)	0.09 (0.10)	0.92 (0.36)
Mainstream VA-B (Alabama)	-	2.96 (0.05)	-0.18 (0.08)	-2.19 (0.03)
Mainstream VA-C (Alabama)	-	2.93 (0.08)	-0.27 (0.10)	-2.73 (0.01)
Health^2^	F = 3.38 (p = 0.07)	-	-	-
Fair/Poor	-	2.99 (0.04)	-0.08 (0.04)	-
Good/Very Good/Excellent	-	3.07 (0.04)	-	-
Depression/Anxiety^3^	F = 1.79 (p = 0.18)	-	-0.03 (0.02)	-
Severe Psychiatric Symptoms^3^	F = 2.39 (p = 0.12)	-	-	-
Absence of symptoms	-	3.07 (0.05)	0.17 (0.08)	-
Presence of symptoms	-	2.99 (0.04)	-	-
Drug Severity^4^	F = 0.81 (p = 0.37)	-	-	-
Absence of symptoms	-	3.05 (0.04)	0.04 (0.05)	-
Presence of symptoms	-	3.01 (0.05)	-	-
Alcohol Severity^4^	F = 0.01 (p = 0.93)	-	-	-
Absence of symptoms	-	3.03 (0.05)	-0.003 (0.05)	-
Presence of symptoms	-	3.03 (0.04)	-	-
Housing Status	F = 2.91 (p = 0.03)	-	-	-
Domiciled	-	3.11 (0.05)	0.03 (0.09)	0.38 (0.71)
Recently homeless	-	3.07 (0.08)	-	-
Chronically homeless	-	3.00 (0.05)	-0.07 (0.09)	-0.087 (0.38)
Chronically and recently homeless	-	2.94 (0.05)	-0.13 (0.09)	-1.52 (0.13)
Perceived Extent of Choice^5^	F = 23.29 (p<0.0001)	-	-	-
Strongly disagree	-	2.74 (0.14)	-0.37 (0.15)	-2.51 (0.01)
Disagree	-	2.80 (0.08)	-0.31 (0.09)	-3.40 (0.01)
Agree	-	3.05 (0.04)	-0.06 (0.06)	-1.05 (0.29)
Strongly agree	-	3.46 (0.05)	0.35 (0.07)	5.07 (p<0.0001)
**Social Support** ^6^	F = 0.77 (p = 0.38)	-	0.01 (0.01)	-
Severe Psychiatric Symptoms x Site	F = 3.61 (p = 0.01)	-	-	-
Tailored Non-VA				
Absence of symptoms	-	3.23 (0.06)	-	-
Presence of symptoms	-	3.06 (0.07)	-	-
Tailored VA				
Absence of symptoms	-	3.00 (0.08)	-0.27 (0.13)	-2.14 (0.03)
Presence of symptoms	-	3.10 (0.07)	-	-
Mainstream VA-A				
Absence of symptoms	-	2.98 (0.07)	-0.34 (0.12)	-2.75 (0.01)
Presence of symptoms	-	3.15 (0.08)	-	-
Mainstream VA-B				
Absence of symptoms	-	3.05 (0.08)	-0.01 (0.12)	-0.06 (0.95)
Presence of symptoms	-	2.88 (0.06)	-	-
Mainstream VA-C				
Absence of symptoms	-	3.08 (0.13)	0.12 (0.17)	0.73 (0.47)
Presence of symptoms	-	2.78 (0.08)	-	-

1. After consideration of persons with missing variables, the final model consists of 355 informative study participants. Overall R-squared for the model shown was 0.36, F = 8.95, *p* ≤0.0001. For variables composed of multiple categories, *P* values reflect type 3 tests of fixed effects.

2. General Health is construed as a 5-step variable (Poor, Fair, Good, Very Good, and Excellent).

3. Depression/Anxiety and Severe Psychiatric Symptoms scores are computed from relevant subscales on the Colorado Symptom Index (see Methods).

4. Drug Severity and Alcohol Severity derived from World Health Organization Alcohol, Smoking and Substance Involvement Screening Test Global Continuum Illicit Drug Use Score and Specific Current Alcohol Involvement Score, respectively.

5. Perceived Extent of Choice derived from 4-point Likert response to the item “I can switch primary care providers if I ask”.

6. Social Support derived from 3 items of the “Strong Ties” scale related to companionship and friendship (see Methods).

### Interaction of severe mental health symptoms by site

The interaction between site and severe psychiatric symptoms was significant (F = 3.61, p = 0.01) indicating that the relationship between severe psychiatric symptoms and primary care experience differed by site of care. To illustrate this, a secondary analysis iterated a general linear model once for persons with presence of severe psychiatric symptoms, and once for persons with absence of severe psychiatric symptoms.

For patients without severe psychiatric symptoms (absence), primary care experience score differences between the sites did not attain significance (F = 2.09, p = 0.09). However, for patients with severe psychiatric symptoms (presence), primary care experience differed significantly by site (F = 5.87, p = 0.0002). These scores were higher at two tailored sites and Mainstream A, but lower at the other two mainstream sites (mean and SE for multivariable-adjusted PCQ-H total score, according to site of care, are shown): Tailored Non-VA 3.06 (0.07), Tailored VA 3.10 (0.07), Mainstream VA-A 3.15 (0.08), Mainstream VA-B 2.88 (0.06), and Mainstream VA-C 2.78 (0.08).

## Discussion

Homeless persons with mental health conditions represent a particularly vulnerable population in which factors conducive to a positive primary care experience remain understudied and yet potentially important to ongoing efforts to foster patient-centered care for vulnerable populations. The present analysis sought to identify factors that predict experience across primary care settings that varied in the degree to which they tailored service delivery for homeless persons. Tailoring ranged from standard mainstream clinics to settings that utilized dedicated, specialized staff, special hours and locations of operation, and unique services such as facilitated access to psychiatric care, food, or shelter. Our initial conceptual model, guided by an existing model of patient perceptions of care, [29] is partially supported by our results. We evaluated whether patient characteristics, social support, extent of choice in providers, and primary care tailoring by site, determined experiences in primary care for homeless persons with mental health conditions. The following factors predicted positive primary care experiences: homeless-specific tailoring, perceived extent of choice in providers, and housing status. We also found a unique interaction effect between severe psychiatric symptoms and site suggesting that the relationship between severe psychiatric symptoms and primary care experiences differed depending on site of care. Some patient characteristics (specifically health, depression/anxiety, severe psychiatric symptoms, drug and alcohol severity), and social support were not significantly associated with the primary care experiences in this population.

Tailored services predicted more positive experiences of primary care among homeless persons with severe psychiatric symptoms. Less favorable experiences of primary care were associated with two mainstream primary care settings. The stronger performance of one mainstream site that had some identifiable aspects of homeless-specific tailoring may be due to that tailoring itself, or to other organizational characteristics that we were unable to measure.

Our findings do suggest an association between primary care experience and degree of service tailoring when the sites are considered along a continuum of service tailoring. Since care service deficits for homeless persons with mental health conditions have been widely documented [1, 41], our findings tend to support what recent demonstration projects have also suggested [17, 24–25]; positive primary care experiences appear to be influenced by the service design characteristics of the agency delivering primary care. While studies have reported on specific sites or programs, to our knowledge, no prior study has incorporated data from mainstream and tailored primary care settings in their analysis of patient care experience. Furthermore, findings for site are patient-centered, being based on person-oriented data, rather than utilization or mortality [42–43].

Patients’ perceived ability to switch providers on demand is the strongest predictor of primary care experience in this analysis. Our finding is particularly interesting due to the nature of this variable; a perceived choice in care calls attention to the importance assigned to flexible and patient-centered care. Moreover, the belief that a patient can exercise such control over their care may be an extension of their experience of their care overall. This underscores the importance of the provider-patient relationship [44], as well as the importance of control where uncertainty to predict or anticipate the availability of resources is commonplace [22].

Our findings for housing status, which was categorically broken into domiciled, recently homeless, chronically homeless, and chronically and recently homeless, illustrates the importance of having stable housing to one’s experience of primary care. This is not surprising given the known association between socioeconomic status and health [45–46]. From our findings we might infer that attainment of a stable domicile is associated with a more favorable primary care experience, compared to the least stable (chronically and recently homeless) arrangement. Unfavorable primary care experiences could occur for various reasons, including transinstitutionalization, and other health complications due to unsheltered lifestyle [47].

The interaction between severe psychiatric symptoms and site of care was not expected. At Tailored VA and Mainstream VA-A sites, presence of severe psychiatric symptoms were associated with a more positive rating of the primary care experience. However at Mainstream VA-B and Mainstream VA-C sites, presence of severe psychiatric symptoms were associated with less favorable rating of the primary care experience. There may be something specific to these populations or site of care with regard to how patients with psychiatric symptoms are received or cared for. Mainstream VA-C served as a regional referral site for inpatient psychiatric care and likely had a particularly severe psychiatric case-mix. Based on discussions with Mainstream VA-C clinicians, patients with past inpatient psychiatric hospitalizations were more likely to stay in the vicinity of Mainstream VA-C. The uniquely low primary care experience scores may reflect a particularly extreme population in terms of psychiatric severity.

Meanwhile, the Tailored Non-VA site offers an obviously strong experience for all clients. Its experience for patients with severe psychiatric symptoms was not low, but it did not attain parity of experience for patients with severe psychiatric symptoms. The similarity of scores across both groups (presence and absence of severe psychiatric symptoms) at the Tailored VA site may reflect the reality that this particular tailored VA program was housed within mental health space where the services for mental care were very tightly integrated. In fact, both Mainstream VA-A and Tailored VA sites are full service health systems where outpatient care is provided alongside the full range of psychiatric services, including inpatient care. This does not apply to the Mainstream VA-B and VA-C sites. Mainstream VA-B lacks inpatient psychiatric care and ordinarily sends patients requiring such care to Mainstream VA-C, roughly 80 miles distant. This pattern of service distribution may have implications for the types of formerly homeless patients who reside in close proximity to each of these two facilities. However, we do acknowledge that these findings, exploratory as they are, primarily invite future research directed at understanding how the presence of severe psychiatric symptoms might influence the experience of primary care relationships and services.

Health status approached but did not attain statistical significance as a predictor of primary care experiences. This aligns with prior studies that have had mixed results among similarly homeless vulnerable populations. Some prior studies have found that better quality of care leads to greater satisfaction, and presumably better health [48]. However, other studies have not observed a significant association between health status and satisfaction with care [49–50].

Although it was important to account for depression and anxiety in this population, our findings hint at a complicated relationship with other aspects of care. Since depression and anxiety are risk factors for non-compliance of medical treatment and diminished health status, it is difficult to account for the myriad of direct and indirect reasons why patient experience is not significant. Perhaps assessing changes in depression or anxiety symptoms rather than a static score of depression/anxiety would predict care experience in a more meaningful way.

Interestingly, social support was not a significant predictor of primary care experience among homeless persons with mental illness. While it was assumed that greater social support could be viewed as resource capital with potential to improve healthcare access and outcomes, [51–52] our measure of social support (companionship ratings per Strong Ties scale) may not capture forms of valued social capital in this sample population (i.e., community residential networks related to severe psychiatric symptoms or veteran status, substance disorder support groups, ACCESS, etc.). For example, a patient’s likelihood of forming a strong relationship in primary medical care appears to have little to do with the quality of companionship they obtain outside of care. Other types of social capital not measured here, including group membership, merit future study.

Limitations to this study include the correlational nature of the analysis, requiring caveats regarding causal reasoning. Additionally, our scoring of severe psychiatric symptoms reflect only a part of the symptom constellations in the diagnosis of mental illness, substance misusing, and PTSD. To avoid misleading diagnostic interpretations, scores are translated into “presence of severe psychiatric symptoms,” or “absence of severe psychiatric symptoms” for our sample population. Moreover, data available to this analysis could not cover all variables anticipated by Sofaer and Firminger's conceptual model (e.g. “social/cultural norms,” “reputation of provider”). A qualitative approach could help explore the subtleties of primary care processes and experiences that may otherwise be lost with limited response criteria. Lastly, four out of five sites were VA, and therefore those lacking financial coverage for care was minimal. Exploration of financial barriers may be an important determinant of experience in other settings.

Given ongoing healthcare reform, there is increasing attention to the care of traditionally underserved populations, to patient-centered care, and to patient perceptions of care. We studied organizational and patient factors that affect perception of care among homeless persons with mental health conditions. Strengths of this study include a strong response rate and use of in-person assessment in an underserved, marginalized population that can be very difficult to interview. Results support the importance of tailored care, perceived choice among providers, and housing status. Although there is no formal definition of tailored primary care, the following service design characteristics are believed to be important: co-location, same-day services, and integration of primary care with mental health and substance disorder services. Continued implementation research that offers insight into *what* service characteristics are valued, as well as *how* those service processes are best delivered is needed. To improve performance on patient experience surveys, organizations may want to deliver services that offer greater flexibility and choice to patients experiencing homelessness.
